# Apixaban Versus Low-Molecular-Weight Heparin for Cancer-Associated Venous Thromboembolism: A Systematic Review and Meta-Analysis

**DOI:** 10.3390/jcm15145341

**Published:** 2026-07-08

**Authors:** Sumit Aggarwal, Vikram Singh, Aayushi Bhasin, Sachit Anand, Heena Tabassum

**Affiliations:** 1 Indian Council of Medical Research, New Delhi 110029, India; drvikramicmr@gmail.com (V.S.); aayushi28bhasin@gmail.com (A.B.); 2 All India Institute of Medical Sciences, New Delhi 110029, India; kanusachit@gmail.com

**Keywords:** cancer-associated thrombosis, apixaban, low-molecular-weight heparin, venous thromboembolism, meta-analysis

## Abstract

**Background**: Venous thromboembolism (VTE), particularly in the context of cancer-associated thrombosis (CAT), is a major cause of morbidity and mortality in patients with malignancy. While apixaban has emerged as a potential alternative to low-molecular-weight heparins (LMWHs), uncertainty remains regarding the consistency of its safety profile across different LMWH agents. **Methods**: This systematic review and meta-analysis was conducted in accordance with PRISMA guidelines. A comprehensive literature search was performed across PubMed, Web of Science, and the Cochrane Central Register of Controlled Trials. **Results**: Six randomized controlled trials were included. Apixaban was associated with a reduced risk of recurrent VTE (RR 0.66, 95% CI 0.45–0.96). However, safety outcomes varied depending on the LMWH comparator. Compared with dalteparin, apixaban was associated with a higher risk of clinically relevant non-major bleeding (CRNMB) (log RR 0.38; 95% CI 0.02 to 0.75), whereas, compared with enoxaparin, it was associated with a lower risk (log RR −0.49; 95% CI −0.96 to −0.02). **Conclusions**: These findings suggest potential differences in safety profiles across individual LMWH agents. However, given the limited number of included trials and clinical heterogeneity, the results should be interpreted cautiously. Further large-scale studies are required to confirm these observations.

## 1. Introduction

Venous thromboembolism (VTE) is a common and serious condition, accounting for the third largest number of cardiovascular deaths [1], encompassing deep vein thrombosis (DVT) and pulmonary embolism (PE) [2]. In patients with malignancy, this condition is referred to as cancer-associated thrombosis (CAT), which carries a significantly higher risk of recurrence and mortality. In India, the incidence of PE ranges from 39 to 115 per 100,000 individuals, while DVT ranges from 53 to 162 per 100,000 individuals [3].

Recent epidemiological studies suggest that the global burden of VTE continues to increase, driven by an aging population, improved detection, and a rising prevalence of risk factors such as malignancy and surgery [4].

Thrombus formation may be initiated by three primary factors: vascular injury, venous stasis, and hypercoagulability, collectively known as Virchow’s Triad [2]. Hypercoagulability is a pathological condition in which excessive coagulation or coagulation without bleeding occurs, which may be induced by malignancy through an activation of the coagulation cascade [5]. Cancer is a substantial risk factor for VTE, accounting for 18% of overall occurrences and being the best predictor of all-cause and PE-related mortality in VTE [6]. The management of CAT is challenging, with an unpredictable risk of developing complications based on patients’ susceptibilities.

CAT treatment involves three phases: the initial or acute phase (5–7 days), the long-term phase (up to 3 months), and the prolonged phase [7]. Anticoagulants have been the primary line of treatment for the past two decades, including heparins/low-molecular-weight heparins (LMWHs), indirect factor Xa inhibitors, and vitamin K antagonists [8]. Currently, the treatment regimen is shifting to DOAC (direct oral anticoagulants) to prevent and treat CAT. Nevertheless, anticoagulants do not dissolve existing clots; instead, they stabilize the thrombus and reduce the risk of new VTE events. There are several drawbacks associated with LMWH, encompassing patient discomfort, expense, absence of a comprehensive antidote, contraindication in severe renal impairment, and thrombocytopenia, raising concerns over both bleeding and thrombosis associated with heparin-induced thrombocytopenia [9].

DOACs, introduced in the mid-2000s, offer the convenience of oral administration without routine monitoring. They have two subgroups: factor IIa (dabigatran) and factor Xa inhibitors (apixaban, edoxaban, and rivaroxaban) [10]. Apixaban (Eliquis), a factor Xa inhibitor with a 30,000-fold higher selectivity for factor Xa, was approved on 28 December 2012 [11]. Apixaban has demonstrated a favorable safety profile in clinical trials compared with other oral anticoagulants [12]. Also, unlike other anticoagulant treatments, apixaban is an easy-to-administer oral tablet predominantly absorbed in the distal small intestine and the ascending colon, making it a suitable option for patients who have undergone a gastrectomy [13].

It is important to recognize that dalteparin, rather than enoxaparin, demonstrated a statistically significant superiority over vitamin K antagonists in landmark trials of CAT [14,15]. As a result, dalteparin became the standard active comparator in most subsequent trials, like Hokusai-VTE and SELECT-D [16,17].

In this systematic review and meta-analysis, we aimed to compare apixaban with individual LMWH agents (dalteparin and enoxaparin) in patients with cancer-associated thrombosis. Specifically, we evaluated differences in efficacy (recurrent VTE) and safety outcomes, including major bleeding and clinically relevant non-major bleeding (CRNMB). Unlike previous meta-analyses that grouped LMWHs (enoxaparin and dalteparin) as a single class, this study explores potential differences between individual agents to better inform individualized treatment decisions.

## 2. Methodology

### 2.1. Study Design

This study was conducted as a meta-analysis of randomized controlled trials (RCTs). The study was conducted to compare the effectiveness and safety of commonly used anticoagulant treatments, DOACs (apixaban) and LMWHs, in treating CAT. This study was conducted in accordance with the PRISMA (Preferred Reporting Items for Systematic Reviews and Meta-Analysis) guidelines. The study protocol was registered on Prospero, with the protocol number CRD420251244203. A prespecified subgroup analysis was conducted to compare and to test for a differential class effect.

### 2.2. Eligibility Criteria

The eligibility criteria were defined according to the PICO element as follows:

Population (P)—adults (≥18 years) with active cancer (any type and stage) or patients with high risk of developing cancer diagnosed with VTE (including DVT and/or PE) where VTE outcomes were reported; Intervention (I)—apixaban used for treatment and/or secondary prevention of cancer-associated VTE; Comparison (C)—other anticoagulants, including LMWH (dalteparin and enoxaparin); Outcomes (O)—primary outcomes: recurrent VTE (DVT or PE), major bleeding; secondary outcomes: CRNMB.

The included studies were only in English. Since apixaban was approved in the USA in 2012, this review included only studies conducted after that year. Only studies that included a head-to-head comparison with LMWHs were included, excluding all other anticoagulants or direct oral anticoagulants. The studies that involved other associated diseases, pregnancy, stroke, myocardial infarction, or arterial embolism were excluded. Furthermore, studies that focused on cost-effectiveness comparisons, placebo comparisons, or dose-range effects were also omitted.

### 2.3. Information Sources

We performed an extensive search across several databases from 2012 to August 2025, including PubMed, Web of Science, the Cochrane Central Register of Controlled Trials, and clinicaltrials.gov.in. Additionally, a manual search was conducted on Google Scholar and various other clinical trial registers for individual countries, such as the Chinese Clinical Trial Registry (ChiCTR), the EU Clinical Trials Register, and the WHO’s International Clinical Trials Registry Platform (ICTRP), to identify any missing trials and gray literature.

The search strategy combined Medical Subject Headings (MeSHs) and free-text terms related to anticoagulants, venous thromboembolism, and cancer. Boolean operators (“AND” and “OR”) were used to optimize retrieval. The detailed search strategy is provided in the Supplementary Materials. The search strategy is presented in Table 1.

Initially, all anticoagulants were included in the search strategy to ensure that no relevant study, including those with multiple drug comparisons, was overlooked. All possible permutations and combinations of search terms were considered. The inclusion criteria for this SRMA required trials with direct, head-to-head comparisons between apixaban and LMWHs. Although the evaluation and screening process was thorough for all LMWHs, only dalteparin and enoxaparin were ultimately included, as clinical trials directly comparing other LMWHs with apixaban and evaluating the required outcomes were not available.

### 2.4. Screening

The title and abstract screening were conducted in Rayyan (https://new.rayyan.ai/, Rayyan Systems Inc., Cambridge, MA, USA) by two independent reviewers, VS (a biotechnologist) and AB (a toxicologist). Each article was marked as “included,” “excluded,” or in the “maybe” category. In case of conflicts and disputes, a third reviewer, SA (a clinician and epidemiologist), assisted, and all conflicts were resolved through internal discussions. Furthermore, all the included articles were individually screened for full text by both reviewers, which determined the final inclusion of the studies.

### 2.5. Data Extraction

Data extraction was independently performed by two reviewers, VS and AB, and discrepancies were resolved through consensus with a third reviewer. The data were compiled into an Excel sheet in the following format: study, author, clinical trial, study center, treatment vs. control, primary outcome, secondary outcome, apixaban dose, and comparator.

### 2.6. Study Risk-of-Bias Assessment

The following six studies were selected as depicted (in Table 2). The risk-of-bias assessment was done using the Cochrane Risk-of-Bias Assessment tool for Randomized trials (ROB-2) [18]. Six studies were assessed across five domains, and a complete analysis is presented in the results section, along with the representation.

### 2.7. Statistical Analysis

The meta-analysis was conducted using STATA version 16 (statistical software for data science). We conducted a quantitative synthesis using standard meta-analytic techniques. Treatment outcomes were assessed by calculating the log risk ratio (RR) and 95% confidence interval for three key outcomes: recurrent VTE, bleeding, and CRNMB. To evaluate heterogeneity among studies, we used the I^2^ statistic, τ^2^, and Cochran’s Q test using a restricted maximum likelihood (REML) random-effects model. We also performed subgroup analyses to explore potential reasons for differences between the two LMWH comparators, dalteparin and enoxaparin. Differences between these subgroups were evaluated with the Q-test. Statistical significance was defined as a two-sided *p*-value < 0.05. To visually represent both individual and pooled effects, forest plots were generated. Publication bias was evaluated using funnel plots for visual inspection. Each outcome was analyzed separately, utilizing individual datasets for recurrent VTE, bleeding, and CRNMB.

### 2.8. Certainty of Evidence Assessment

The certainty of evidence assessment for each evidence was assessed using the Grading Of Recommendations Assessment, Development and Evaluation (GRADE) framework. The certainty of evidence is evaluated across five domains: risk of bias, inconsistency, indirectness, imprecision, and publication bias. This study includes RCT studies, initially rated at higher certainty of evidence and downgraded in cases where concerns were identified. Gradepro GDT (https://www.gradepro.org/, McMaster University and Evidence Prime Inc., Hamilton, ON, Canada) [25] software was used to generate the Summary of Outcomes findings (SOF).

## 3. Results

### 3.1. Study Selection

**Figure 1:**
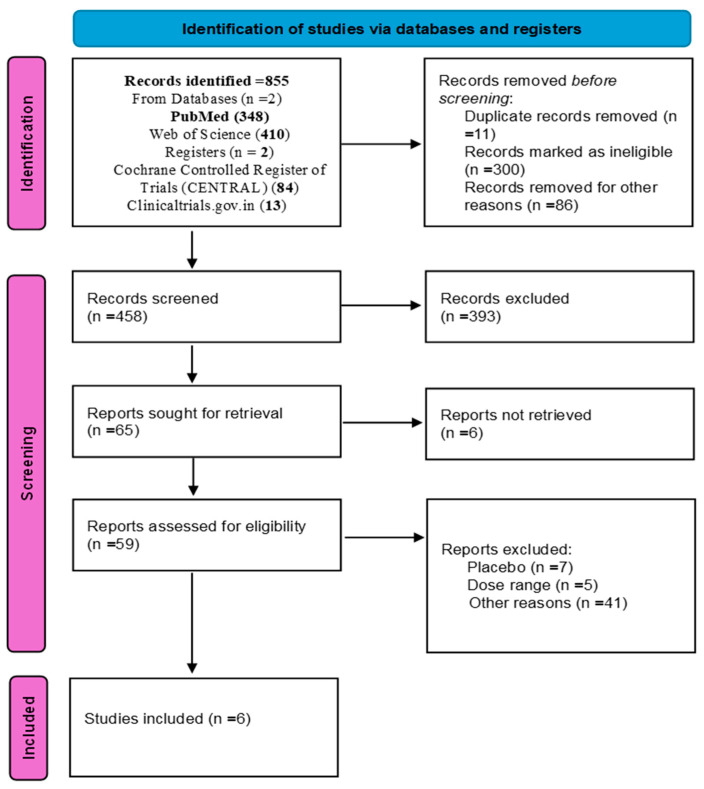
The PRISMA flow diagram.

An extensive search across four identified sources resulted in 855 papers across two databases, PubMed (348) and Web of Science (410), and two registers, CENTRAL (84) and Clinicaltrials.gov.in (13). After removing 11 duplicates, 844 unique records remained. Of these, 300 articles were excluded due to unavailable full text or ineligible publication types, and 86 were excluded due to incomplete metadata or non-English language. A total of 458 records underwent title and abstract screening, of which 393 were removed based on PICOS eligibility criteria. Around 180 observational studies, 100 review articles, and 70 case reports and case series were removed, while 43 articles had different interventions or outcomes. Subsequently, 65 reports were assessed for full text screening, of which six studies were inaccessible. Of the remaining 59 studies, 53 were excluded because they were placebo-controlled trials, comparative dose-ranging regimens, or irrelevant outcomes. Finally, only six RCT studies met our eligibility criteria and were included in the meta-analysis and data synthesis (Figure 1). The screening was conducted using Rayyan software.

The study objective was to compare the safety and efficacy outcomes of apixaban, a DOAC, with those of other LMWHs, including dalteparin and enoxaparin. We selected six randomized controlled trials including 2170 patients with cancer-associated VTE. The LMWHs were further subgrouped as apixaban vs. dalteparin (three studies, 1501 patients, [19,20,21] and apixaban vs. enoxaparin (three studies, 669 patients [22,23,24]).

### 3.2. Sociodemographic Characteristics

Across the six included RCTs, several similarities were observed alongside methodological differences, particularly in dosage regimens, patient populations, and geographic variation. All six studies reported varied sample sizes, ranging from 59 [21] to 1155 patients [20]. The median age ranged from 58 to 67 years, indicating an older adult population across trials. The age distribution was well balanced between the intervention (apixaban arm) and the comparator arm, suggesting similar baseline characteristics across the arms. The baseline male-to-female distribution was comparable between the intervention and comparator groups (Table 2). There was a slight male predominance in all the trials, except for a study [23] which included only a female population. The proportion of male participants ranged from 40% to 56.8% in the apixaban arm and 43.2% to 56% in the comparator arm. The included trials were conducted across diverse settings, including the US, Republic of Korea, and multicenter and multinational cohorts. This geographical variation may enhance the generalizability of the findings across different healthcare settings.

### 3.3. Treatment Characteristics

#### 3.3.1. Dosage

Five [19,20,21,23,24] of the six included studies used a standardized apixaban regimen (10 mg twice daily for 7 days, followed by 5 mg daily thereafter for the remaining treatment duration). The dalteparin arm used the same dose of subcutaneous dalteparin (200 IU/kg for 1 month, followed by 150 IU/kg once daily), consistent with the international standard for LMWH therapy in cancer [19,20]. In contrast, enoxaparin dosing varied across studies, reflecting the absence of a uniform protocol.

#### 3.3.2. Treatment Duration and Follow-Up

The treatment follow-up duration was predominantly around six months across most trials (five out of six trials [19,20,21,22,24] aligned with the standard treatment duration for cancer-associated VTE treatment), while one trial reported a 90-day follow-up period [23]. In the study by Agnelli et al. [20], a 20-day follow-up was conducted for early events, followed by a 6-month extended follow-up (Table 3).

#### 3.3.3. Diagnostic Techniques

Across the six included trials, VTE was confirmed using standardized imaging modalities. The diagnosis of deep vein thrombosis (DVT) was confirmed using various techniques, including duplex ultrasonography, venography, computed tomography (CT) [21], and magnetic resonance imaging (MRI), with the clinical pre-test probability assessed using the Wells’ criteria [23]. Pulmonary embolism (PE) was diagnosed using computed tomography pulmonary angiography (CTPA) [20], magnetic resonance angiography [20], or ventilation–perfusion (V/Q) imaging [19].

### 3.4. Disease Characteristics

#### 3.4.1. Malignancy Sites

The six included studies show a wide range of malignancies, reflecting the real-world heterogeneity in cancer-associated VTE (Table 3). The most common types of cancer across the trials were gastrointestinal (colorectal—160, gastric/upper GI—90, pancreatic/hepatobiliary—100), lung cancer (18–22%), gynecological (500), breast (13–15%), genitourinary (approx. 100), and hematologic (7.4%).

#### 3.4.2. Cancer Stage

In the six included RCTs, most of the patients demonstrated higher or metastatic stages of cancer, highlighting a higher risk of VTE. In the apixaban vs. dalteparin subgroup, the study by McBane et al [19] reported that 65.3% of patients in the apixaban arm and 66.0% in the dalteparin arm had metastatic disease. The study by Agnelli et al. [20] found that approximately 70% of patients had metastatic cancer. The study by Kim et al. [21] reported advanced cancer in 86–88%; most patients reported stage III–IV, metastatic cancer. For the apixaban vs. enoxaparin subgroups, the study from Agnelli et al [22] found that approximately 33% of patients demonstrated metastatic disease and 3.1% showed active cancer. In the study Guntupalli et al. [23], 39.2–43.4% had low-stage disease (I, II), while 37.8–41.2% had high-stage disease (III, IV). Ref. [24] predominantly reported stage IV disease (80–88%) (Table 3).

#### 3.4.3. VTE Presentation

The VTE index varied across the six included trials, with reports of DVT, PE, or combined presentations. In the apixaban vs. dalteparin arm [19], the initial events were PE alone (43.5%), PE with DVT (11.6%), and isolated DVT (36.7%). The lower limbs were mostly affected (31–34%), while the upper extremities (14–17%), the splanchnic region (8–18%), and cerebral venous thrombosis (≤1.4%) were less frequent. The CARAVAGGIO trial [20] also showed a similar balance between symptomatic or incidental PE and proximal DVT, consistent with its inclusion of various cancer types. Kim et al. [21] focused on upper gastrointestinal and hepatopancreatobiliary cancers, marking events of PE dominant in both groups, particularly among those receiving direct oral anticoagulants (DOACs) (79.5% vs. 69.6% for dalteparin), with the remainder attributable to DVT.

Similarly, in studies comparing apixaban to enoxaparin, the VTE patterns were similar [22] reported approximately twice as many DVTs as PEs, with most DVTs proximal (79–82%) and most PEs classified as moderate or extensive. In the study conducted by Guntupalli et al. [23], which focused on gynecologic cancers, the number of VTE events was low (apixaban: two PEs; enoxaparin: three DVTs). Mokadem et al [24] reported that iliofemoral and femoropopliteal DVTs were the most frequent, with similar rates in both the apixaban and enoxaparin groups. Upper-limb DVTs and related thrombophlebitis were rare in both arms (Table 3).

### 3.5. Outcomes

#### 3.5.1. Efficacy Outcome (Recurrent VTE)

In the apixaban intervention group, 3.8% of patients reported recurrent VTE, compared with 6.3% in the comparator group receiving LMWH. The incidence of VTE recurrence in the apixaban group ranged from 0.6% to 5.6%, indicating a lower recurrence rate than in the LMWH group, which ranged from 2.2% to 9.7%. Apixaban showed numerically lower or comparable rates of recurrent VTE.

#### 3.5.2. Safety Outcome (Bleeding)

Gastrointestinal bleeding and mortality events were also reported as composite outcomes in some studies; however, a few did not. The CARAVAGGIO trial [20] reported gastrointestinal bleeding in 1.9% of patients on apixaban and 1.7% on dalteparin. Additionally, the overall bleeding event rate was 12.73% in the apixaban group and 12.15% in the LMWH group, and major bleeding was lower with apixaban than with LMWHs (dalteparin and enoxaparin) across multiple trials.

#### 3.5.3. Mortality

Mortality rates were comparable between the intervention and comparator groups. All-cause mortality was inconsistently reported and showed no statistically significant difference. CARAVAGGIO [20]: 23.4% mortality in Apixaban vs. 26.4% in dalteparin. ADAM VTE [19]: 16% vs. 11%, respectively.

### 3.6. Risk-of-Bias Assessment

The risk-of-bias assessment was conducted using the ROB-2 tool, which is organized into five fixed domains, each focusing on different aspects of trial design. Domain 1 evaluates the risk of bias arising from randomization processes; Domain 2 addresses the risk of bias due to deviations from the intended interventions, specifically the effects of assignment to intervention; Domain 3 examines the risk of bias due to missing outcome data; Domain 4 assesses the risk of bias in the measurement of outcomes; and Domain 5 considers the risk of bias in the selection of reported results. The data were extracted manually from the available journal articles. Additional information was obtained from the non-commercial trial registry record (e.g., ClinicalTrials.gov) and the trial protocol, ensuring no vital information was missed. The plot was generated using the web app—Risk-of-Bias Tools (Robvis) [26]. This visualization tool utilized an Excel sheet to indicate low risk, some concerns, and high risk for five domains for each included study. All the analysis was conducted in an Excel sheet that listed all six studies and assessed the five respective domains using low, high, and some concerns risk scores. After completion, the Excel sheet was uploaded to the Robvis tool to generate a summary plot for risk-of-bias assessment.

**Figure 2:**
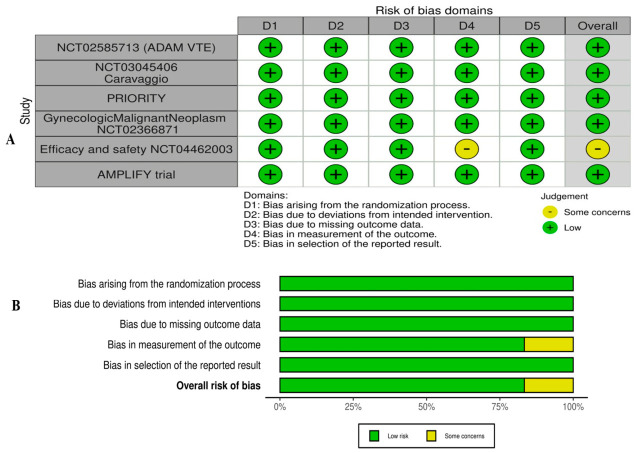
Risk-of-bias assessment: (**A**) study plot and (**B**) summary plot generated by the RobVis tool.

This analysis included six studies spanning five domains. According to Rob-2 analysis, five studies exhibited low risk, while only one study raised concerns across Domain 4, i.e., risk in the measurement of outcome. All other domains were consistent throughout all six studies (Figure 2).

The overall risk-of-bias analysis found the overall risk to be low, and only one study reported some concerns regarding the measurement of the outcome (D4), primarily due to potential limitations in the outcome assessment methods. However, this does not potentially pose a risk to the overall judgment. This study demonstrated a low risk of bias overall in all five domains.

## 4. Meta-Analysis

This meta-analysis was conducted using STATA, focusing on six studies that compared apixaban with two LMWHs. The analysis was stratified by a comparator (dalteparin vs. enoxaparin) into two subgroups. In the first subgroup, which compared apixaban to dalteparin, 734 patients received apixaban and 767 received dalteparin. In the second subgroup, comparing apixaban to enoxaparin, 342 patients received apixaban and 327 received enoxaparin. This study used the log risk ratio (RR) to measure outcomes and a random-effects REML model stratified by comparator (dalteparin vs. enoxaparin). However, interpretation is critical due to the limited number of included trials and heterogeneity across patient populations.

### 4.1. Bleeding Events

A subgroup analysis was conducted to compare the bleeding risk associated with apixaban across the dalteparin and enoxaparin subgroups.

#### 4.1.1. Apixaban vs. Dalteparin

**Figure 3:**
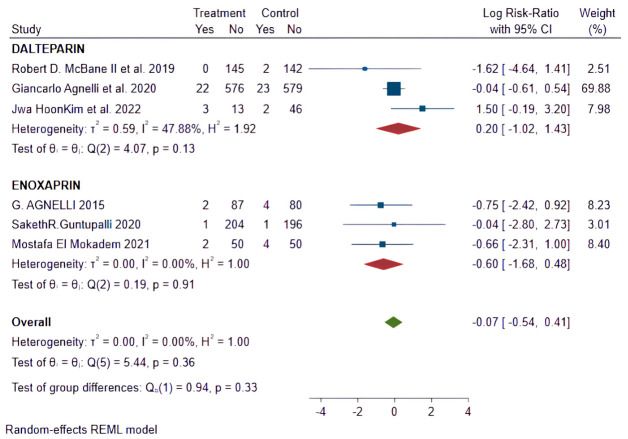
Forest plot for meta-analysis to assess the safety outcome (bleeding) (Blue square = effect size (log risk ratio) of each study; Size of square = study weight (larger = more influence); Horizontal blue line = 95% confidence interval; Red = Subgroup pooled estimate; Green = Overall pooled estimate) [19,20,21,22,23,24].

In the subgroup comparing apixaban with dalteparin, three studies were included: [19,20,21]. McBane et al [19] reported no events in the apixaban arm (0/145) and two bleeding events in the LMWH group (2/142), with a log RR of approximately −1.62 (95% CI: −4.64 to 1.41) and a study weight of 2.51%. Agnelli et al [20] contributed the largest weight (69.88%) and observed 22 events in the apixaban arm (22/576) versus 23 in the dalteparin arm (23/579), with a log RR of −0.04 (95% CI: −0.61 to 0.54). Kim et al. [21] found three events among 13 treated patients and two events among 46 control patients (log RR: 1.50; 95% CI: −0.19 to 3.20; weight: 7.98%). The overall pooled analysis for all three studies shows a non-significant effect (log RR: 0.20; 95% CI: −1.02 to 1.43), suggesting no significant difference in bleeding outcomes between apixaban and dalteparin. Moreover, there was substantial heterogeneity (I^2^ = 47.88%, τ^2^ = 0.59, H^2^ = 1.92; Q (2) = 4.07, *p* = 0.13), suggesting a moderate variability in treatment effects across studies (Figure 3). These findings suggest no significant difference in overall bleeding risk between apixaban and dalteparin. The observed variability across studies may reflect differences in patient population and cancer characteristics.

#### 4.1.2. Apixaban vs. Enoxaparin

In the subgroup comparing apixaban with enoxaparin, three studies were included [22,23,24]. Agnelli et al. [22] reported 2/87 bleeding events in apixaban as compared to 4/80 in the enoxaparin group, presenting a weight of 8.23% (log RR: −0.75; 95% CI: −2.42 to 0.92). Another study, Mokadem et al. [24] reported (2/50) events in apixaban and (4/50) in enoxaparin, which presented a weight of 8.40% (along with log RR: −0.66; 95% CI: −2.31 to 1.00). Guntupalli et al. [23] is the smallest study, with the smallest weight (3.01%) (log RR: −0.04; 95% CI: −2.80 to 2.73).

The pooled analysis of enoxaparin studies found no significant difference in bleeding risk between apixaban and enoxaparin (log RR: −0.60; 95% CI: −1.68 to 0.48). Despite the differences in sample sizes and incident rates, enoxaparin studies suggest highly consistent results, with no heterogeneity (I^2^ = 0.00%, τ^2^ = 0.00, H^2^ = 1.00; Q (2) = 0.19, *p* = 0.91) (Figure 3).

### 4.2. Clinically Relevant Non-Major Bleeding

#### 4.2.1. Apixaban vs. Dalteparin

**Figure 4:**
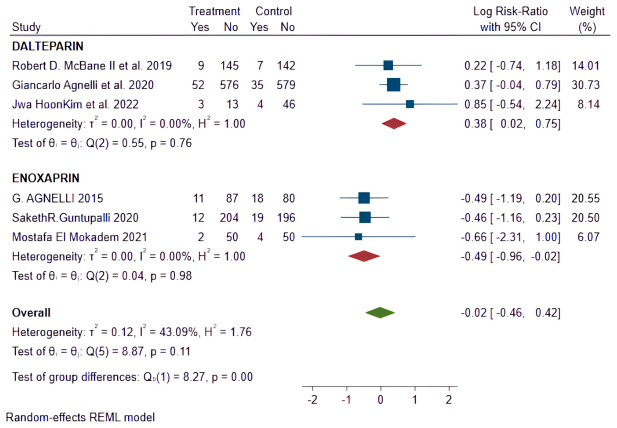
Forest plot for meta-analysis of CRNMB [19,20,21,22,23,24].

In the dalteparin subgroup, we investigated three studies [19,20,21]. The pooled analysis (log RR ≈ 0.38 (95% CI ~0.02–0.75)) suggests that apixaban is associated with an increased risk of CRNMB compared to dalteparin. This corresponds to an RR of approximately 1.46 (95% CI 1.02 to 2.12), indicating a 46% higher risk of CRNMB with apixaban. No significant heterogeneity was observed among dalteparin studies (I^2^ = 0%, τ^2^ = 0.00, H^2^ = 1.00; Q (2) = 0.55, *p* = 0.76), indicating consistent findings across the included trials. The study Agnelli et al. [20] with the largest population contributed 30.73% and showed a borderline significant effect, while studies from Kim et al., 2022 [21] and McBane et al., 2020 [19] weighed 8.14% and 14.01%, presenting non-significant trends in the same direction (Figure 4).

#### 4.2.2. Apixaban vs. Enoxaparin

In the three included studies [22,23,24], the pooled analysis suggests that apixaban was associated with a statistically significant lower risk of CRNMB compared to enoxaparin (log RR −0.49; 95% CI −0.96 to −0.02, *p* = 0.04). This corresponds to a relative risk of approximately 0.61 (95% CI 0.38 to 0.98), indicating a 39% lower risk of CRNMB with apixaban compared to enoxaparin. No measurable heterogeneity (I^2^ = 0.00%, τ^2^ = 0.00, H^2^ = 1.00; Q (2) = 0.04, *p* = 0.98) was detected among enoxaparin group. Notably, Saketh R. Guntupalli et al [23] and Mostafa El Mokadem et al [24] contributed effects in the same direction, with Mokadem et al. [24] contributing 6.07% weight, reinforcing the same conclusion, indicating the effectiveness of enoxaparin over apixaban (Figure 4 ).

#### 4.2.3. Overall Analysis

All six studies when combined present a non-significant pooled effect estimate (log RR ≈ −0.02; 95% CI: −0.46 to 0.42), suggesting no significant difference between the intervention and comparator groups when analyzed as a class, which significantly masks the individual findings. There is heterogeneity in the overall findings (I^2^ = 43.09%, τ^2^ = 0.12, H^2^ = 1.76; Q (5) = 8.87, *p* = 0.11), which may reflect opposing effects of dalteparin and enoxaparin. The test for subgroup differences was statistically significant (Q (1) = 8.27, *p* = 0.00), indicating a substantial and meaningful difference between the two LMWHs. This suggests that individual LMWH agents may exhibit differential safety profiles, warranting further future research.

### 4.3. Recurrent VTE

#### 4.3.1. Apixaban vs. Dalteparin

**Figure 5:**
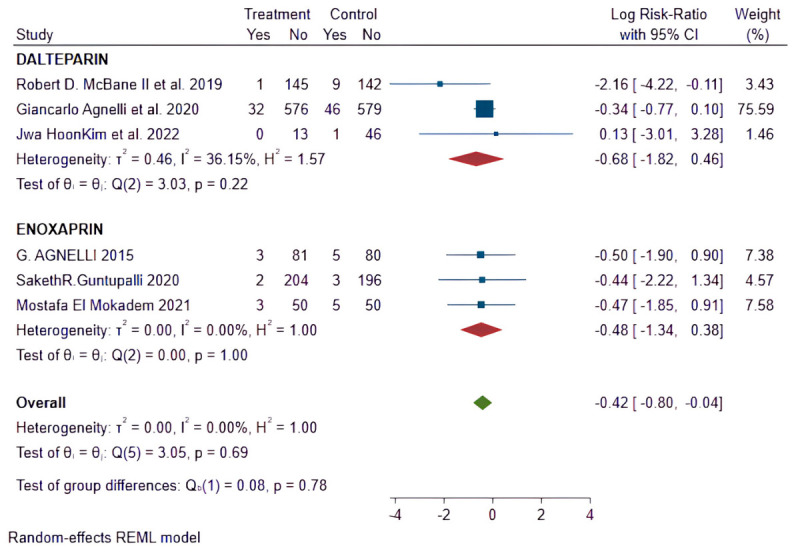
Forest plot for meta-analysis of six studies to assess efficacy outcome (recurrent VTE) [19,20,21,22,23,24].

In this subgroup, three studies were included [19,20,21]. The pooled log RR = −0.68 (95% CI: −1.82 to 0.46), indicating a non-significant reduction in recurrent VTE with apixaban compared to dalteparin. Moderate heterogeneity was observed (I^2^ = 36.15%, τ^2^ = 0.46; Q (2) = 3.03, *p* = 0.22), suggesting some variability across studies and a non-significant reduction in recurrent VTE (Figure 5).

#### 4.3.2. Apixaban vs. Enoxaparin

In this subgroup, three studies were included [22,23,24]. The pooled log risk ratio was −0.48 (95% CI: −1.34 to 0.38), suggesting a statistically non-significant difference in recurrent VTE between apixaban and enoxaparin. No heterogeneity was detected (I^2^ = 0%), indicating consistent findings across studies (Figure 5).

## 5. Overall Analysis

The pooled analysis of six RCTs suggested that apixaban is associated with a statistically significant reduction in recurrent VTE compared with LMWHs overall (log RR = −0.42; 95% CI: −0.80 to −0.04). No heterogeneity was observed (I^2^ = 0%). The test for subgroup differences was statistically non-significant (Q (1) = 0.08, *p* = 0.78), suggesting that the treatment effect did not differ significantly between dalteparin and enoxaparin (Figure 5).

### 5.1. Risk Assessment of Publication Bias

Three funnel plots were generated for our three specified outcomes: safety (bleeding and CRNMB) and efficacy (recurrent VTE). The plots were generated in R to evaluate potential publication bias in the studies’ reporting of outcomes. Each point in the plot represents an individual study, with the standard error plotted against the risk ratio.

#### 5.1.1. Bleeding

A funnel plot analysis was performed to evaluate potential publication bias in studies assessing bleeding outcomes. The vertical dotted line at the center represents log RR = 0, suggesting no treatment effect, while the triangles on either side denote the 95% pseudo confidence limits. The horizontal axis displays the logarithm of the risk ratio, with 0 indicating no effect. Most studies fall within the 95% pseudo-CI, with one study located slightly towards the lower left, suggesting a larger standard error or imprecision, possibly due to a smaller sample size. No apparent funnel asymmetry or pronounced clustering on either side is observed, suggesting no substantial publication bias for bleeding outcomes. However, since only six RCTs were included, the funnel plot’s ability to reliably detect asymmetry is limited (refer to Supplementary Materials for plot).

#### 5.1.2. CRNMB

A funnel plot analysis was performed to assess potential publication bias in the studies’ CRNMB outcomes. The most significant and most precise studies appear symmetrically at the top of the plot, clustered near the pooled estimate (red vertical line). Smaller studies are distributed toward the bottom on both the right and left sides, reflecting increased variability, typically observed in trials with smaller sample sizes. The funnel plot for the CRNMB outcome demonstrates overall symmetry. These findings suggest no significant publication bias or small-study effects for the CRNMB outcome. However, given the limited number of RCTs, subtle bias cannot be entirely excluded (refer to Supplementary Materials for plot).

#### 5.1.3. Recurrent VTE

A funnel plot analysis was conducted to evaluate potential publication bias in the studies’ efficacy outcomes. The top two studies are most precise and very close to the pooled estimate. At the bottom of the funnel, two studies indicate asymmetry: one on the right and one on the left. However, both studies fall within the 95% pseudo-confidence limits. The plot suggests an asymmetry more consistent with random variability across smaller studies than with publication bias. However, small-study effects cannot be excluded with certainty, given the small number of trials. Additionally, Egger’s test could not be performed, as it requires at least 10 studies (refer to Supplementary Materials for plot).

### 5.2. Certainty of Evidence (GRADE Assessment)

**Figure 6:**
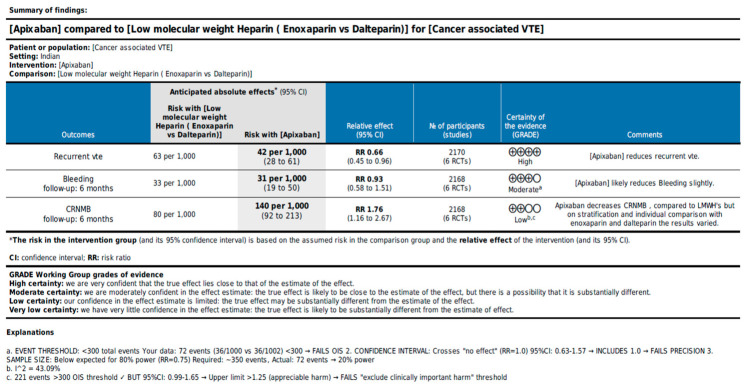
Grade SOF (Summary of Outcomes finding) table (Certainty of evidence was assessed using the GRADE approach, where: ⊕⊕⊕⊕ indicates high certainty of evidence, ⊕⊕⊕◯ indicates moderate certainty, ⊕⊕◯◯ indicates low certainty.

The certainty of evidence assessment was performed individually for each of the three outcomes of interest: recurrent VTE, bleeding, and CRNMB. As all the included studies were RCTs, the quality of evidence was initially rated high and subsequently downgraded based on domain-specific concerns. The primary efficacy outcomes did not raise serious concerns across all five domains, with the recurrent VTE outcome graded as high. The pooled estimates were consistent, with narrow confidence intervals and no observed heterogeneity supporting a reliable effect estimate. Regarding bleeding outcomes, the certainty of evidence was moderate, with some concerns in the imprecision domain, downgrading it to moderate due to the small number of events and wide confidence intervals that often cross the line of no effect. While these studies were well designed and low in bias, the small sample sizes make it harder to be certain about the true effect size. In the CRNMB outcome, there were a few concerns across two domains, serious inconsistency and imprecision due to moderate heterogeneity, and a wide confidence interval, which reduced the precision of the estimate and led to a low rating of the evidence. Overall, the evidence strongly supports the recurrent VTE outcome; discrepancies may be warranted in bleeding and CRNMB outcomes due to variability across studies and limited precision. The assessment was conducted manually on the Grade Pro GDT [25], and a Summary of Outcomes (SOF) table was generated (Figure 6).

## 6. Discussion

This systematic review and meta-analysis provides a comparative evaluation of apixaban and individual LMWH agents (dalteparin and enoxaparin) in the management of cancer-associated venous thromboembolism. While previous meta-analyses have typically grouped LMWHs as a single class, this study explores potential differences between individual agents.

The study findings suggest that apixaban may be associated with improved efficacy compared with LMWHs. This study suggests a potentially important finding, particularly regarding CRNMB, which is not uniform across dalteparin and enoxaparin. This observation challenges the “LMWH class effect”, while our findings align with the previous evidence [27,28,29]. This study identifies a potential gap in the literature and warrants further future research on a characterized cancer population and may further provide a framework for individualized treatment based on cancer type and individual susceptibilities.

In the stratified analysis by subgroup, the apixaban vs. dalteparin arm shows apixaban’s efficacy in reducing the risk of recurrent VTE. Meanwhile, in the apixaban vs. enoxaparin subgroup, there was a statistically significant reduction in recurrent VTE, favoring apixaban. These findings align with large trials such as [20] (CARAVAGIO trial), which demonstrate the non-inferiority of apixaban in reducing the risk of recurrent VTE, and [22] (AMPLIFY trial), which indicates comparable efficacy between apixaban and enoxaparin. Several meta-analyses also reported consistent findings [27,28], highlighting the efficacy of apixaban compared with LMWHs. A major challenge in the management of CAT is the risk of bleeding. This SRMA reported no significant difference in overall bleeding rates between apixaban and LMWHs. These results support a previously conducted meta-analysis, which reported no significant difference between apixaban and LMWHs in terms of total bleeding (RR: 1.16; 95% CI (0.91to 1.48); *p* = 0.23; I2 = 0% [28]). Also, these results were consistent with large trials [20,22] (CARRVAGIO and AMPLIFY trials), supporting overall comparative safety. However, a smaller trial (n = 13 for apixaban) [21] (PRIORITY trial) reported that apixaban was associated with a notably higher bleeding rate, suggesting heterogeneity between the trials. The inconsistencies may be a result of the cancer type (upper gastrointestinal and hepatopancreatobiliary malignancies), which may be associated with a higher bleeding risk.

These findings on preventing recurrent VTE and bleeding in the CAT population align with previous meta-analyses regarding DOACs used in acute and cancer-associated VTE settings [29]. The study analysis indicates that, compared to dalteparin, apixaban was associated with a significantly higher risk of CRNMB (log RR 0.38; 95% CI 0.02 to 0.75), corresponding to a 46% increased relative risk. In contrast, apixaban was associated with a significantly lower risk of CRNMB compared with enoxaparin (log RR = −0.49; 95% CI = −0.96 to −0.02). This differential effect was statistically robust (*p* for subgroup difference = 0.004) and remained consistent across the included trials. The observed differences in CRNMB between dalteparin and enoxaparin suggest a possible variation within the LMWH class. Although these findings are consistent with earlier studies [30], they should be interpreted with caution due to the limited number of included trials and wide confidence intervals, and warrant confirmation in future studies.

This finding challenges the assumption of the LMWH class effect and suggests individualized future research to ensure a strong safety profile for apixaban to replace LMWHs in CAT. This may warrant consideration in future research and guidelines updates.

A study [20] (CARRAVAGIO trial) mentioned gastrointestinal bleeding as one of the most significant bleeding events in cancer. The bleeding rates with apixaban and dalteparin were 1.9% and 1.7%, respectively, suggesting that apixaban does not substantially increase the risk of gastrointestinal bleeding in cancer patients. The CARRAVAGGIO trial [20] reported mortality of 23.4% in the apixaban arm versus 26.4% in the dalteparin arm, and the ADAM VTE trial [19] reported mortalities of 16% versus 11%, respectively. The absence of a statistically significant difference in mortality may reflect the underlying progression of malignancy rather than treatment effects.

This study reports on a wide variety of cancers (gynecological cancers, mixed solid tumors, gastrointestinal cancers, lung, pancreatic, and hepatobiliary cancers). Although this reflects a real-world scenario, it also introduces potential confounding, such as variation in bleeding and thrombotic risks. Gastrointestinal tract and hepatopancreatobiliary cancers were found in 30–35% of patients, accompanied by a high bleeding risk. The study by Kim et al. [21], which reported only on upper gastrointestinal and hepatopancreatobiliary cancers, found the highest bleeding rate in patients taking apixaban (23.1%), much higher than in other trials. Most of the studies reported advanced-stage or metastatic cancer, with metastasis ranging from 33 to 88 (PRIORITY trial). Kim et al [21] reported the highest proportion of advanced disease (86–88%), while Guntupalli et al. [23] reported a balanced distribution between early stages (39–43%) and advanced-stage disease (38–41%). The indexed VTE events varied considerably across trials, with DVT, PE or combined presentations. A study explored the combined use of palliative chemotherapy and anticoagulant therapy [21]. Chemotherapy can complicate the assessment of thrombotic and bleeding risks, as it influences these outcomes through multiple pathways, raising the risk of bleeding, and may shift the balance of risks and benefits when using anticoagulants. Geographical variations may include confounders such as differences in genetic factors, patient populations, healthcare systems, and clinical practices.

The overall quality of evidence varied by outcomes, suggesting a high certainty for recurrent VTE, moderate certainty for bleeding, and low certainty for CRNMB. The strengths of the study lie in its rigorous methodology, in accordance with the PRISMA statement for SRMA. This study included only RCTs, thereby providing high-quality evidence for clinical decisions and the stratification of LMWHs, and for conducting individual subgroup analyses, comparing the two comparators with the intervention, assessing multiple outcomes, and conducting risk-of-bias and publication-bias assessments, along with a certainty-of-evidence assessment using GRADE.

These findings suggest that apixaban may represent a potential alternative for cancer-associated VTE, with the advantages of easy administration and low monitoring needs. However, various studies have grouped overall LMWHs as a class, neglecting the possible individual differences and variations in overall drug safety. The contradictory CRNMB events in LMWHs (enoxaparin and dalteparin) vs. apixaban highlight the need to synthesize stronger evidence to establish apixaban’s overall safety as a viable alternative to LMWHs. There is a strong need for future research in this area, requiring high-quality clinical trials that focus on similar safety and efficacy outcomes to address many unanswered questions about this critical issue in the current treatment landscape for specific subpopulations, particularly those with cancer-associated VTE and various malignancy types.

While these findings provide important insights, they should be interpreted in the context of certain inherent limitations. The primary limitation of the study is the limited overall body of evidence, with only a small number of clinical trials comparing apixaban with individual LMWHs. The small number of included trials and variability in study design may limit the robustness of subgroup analyses.

To ensure a comprehensive systematic analysis of evidence, two trials with methodological differences were included: Guntupalli SR et al. [23] and Kim et al. [21]. While these studies are not traditional head-to-head randomized trials comparing apixaban with LMWHs in patients with CAT, they provide important insights by involving relevant cancer populations and reporting meaningful outcomes for VTE and bleeding. Upon removal of these studies, the evidence base will become smaller, with weaker subgroup analyses. To minimize potential bias, a subgroup analysis was conducted with a discussion of methodological differences. Furthermore, the certainty of our findings for CRNMB is limited by imprecision and the small number of available trials. This necessitates a cautious interpretation of the results. Additional limitations include the clinical heterogeneity across included cancer types and disease stages, variability in the use of concomitant anticancer therapies, and the incorporation of both a primary prevention trial [23] and a three-arm trial with a relatively small apixaban cohort [21]. Sensitivity analyses excluding the Guntupalli trial did not substantially alter the results, suggesting robustness to this source of heterogeneity. Also, in reporting outcomes, specifically bleeding, some trials did not report CRNMB separately from major bleeding, and gastrointestinal bleeding was not consistently distinguished from bleeding at other sites. We acknowledge these limitations and encourage readers to interpret our findings in light of these factors. These findings should therefore be considered exploratory and hypothesis-generating rather than definitive.

## 7. Conclusions

This study suggests that apixaban may be an effective and convenient treatment option for cancer-associated VTE; however, its safety profile may vary depending on the LMWH comparator. The findings highlight the importance of individualized treatment decisions based on patient characteristics and cancer type. Given the limited number of trials and observed heterogeneity, these results should be interpreted with caution, and further high-quality, adequately powered studies are needed to confirm these findings.

## Figures and Tables

**Table 1:** Search strategy used for database retrieval.

**PubMed**	
**Anticoagulant (Intervention/Comparator)**	Apixaban”[Mesh] OR Apixaban[Tiab] OR Eliquis[Tiab]) AND (“Anticoagulants”[Mesh] OR Anticoagulant*[Tiab] OR “Low Molecular Weight Heparin”[Mesh] OR LMWH [Tiab] OR Dalteparin[Tiab] OR Enoxaparin[Tiab] OR “Vitamin K Antagonists”[Mesh] OR warfarin[Tiab] OR “Direct Oral Anticoagulants”[Mesh] OR DOAC*[Tiab] OR Rivaroxaban[Tiab] OR edoxaban[Tiab]
Condition	(“Venous Thromboembolism”[Mesh] OR “venous thromboembolism”[tiab] OR VTE [tiab] OR “deep vein thrombosis”[tiab] OR DVT [tiab] OR “pulmonary embolism”[tiab] OR PE [tiab])
Population	(“Neoplasms”[Mesh] OR cancer[tiab] OR malignancy[tiab] OR malignancies[tiab] OR neoplasm*[tiab] OR tumor*[tiab] OR tumour*[tiab]) on pubmed.
**Web of Science**	
**Intervention**	**TS = (‘Apixaban’ OR ‘Eliquis’)**
**Comparator**	TS = (‘anticoagulant*’ OR ‘Low Molecular Weight Heparin’ OR ‘LMWH’ OR ‘dalteparin’ OR ‘enoxaparin’ OR ‘Vitamin K Antagonists’ OR ‘warfarin’ OR ‘Direct Oral Anticoagulants’ OR ‘DOAC*’ OR ‘rivaroxaban’ OR ‘edoxaban’)
**Condition**	TS = (‘venous thromboembolism’ OR ‘VTE’ OR ‘deep vein thrombosis’ OR ‘DVT’ OR ‘pulmonary embolism’ OR ‘PE’)
**Population**	TS = (‘cancer’ OR ‘malignancy’ OR ‘malignancies’ OR ‘neoplasm*’ OR ‘tumor *’ OR ‘tumour*’)

**Table 2:** Table shows the sociodemographic characteristics.

Author	Study Design	Age (Median or Range) Apixaban	Age (Median or Range) Comparator	Male/Female Intervention	Male/Female Comparator	No. of Patients	Apixaban (No. of Patients)	Comparator (No. of Patients)	Intervention (Comparator Group)
Robert D. McBane II et al. [19]	RCT	64.4	64	67 M/78 F (52%)	73 M/69 F (51%)	287	145	142	Dalteparin
Giancarlo Agnelli et al. [20]	RCT	67.2 ± 11.3	67.2 ± 10.9	309 M/267 F	319 M/260 F	1155	576	579	Dalteparin
Jwa Hoon Kim et al. [21]	RCT	64.0 ± 9.5	63.0 ± 9.0	25 (56.8%) M/19 (43.2%)	23(50%) M/23(50%) F	59	13	46	Dalteparin
G. AGNELLI [22]	RCT	65.5	65.1	56.8% M/43.2%F	60.5% M/39.5% F	169	88	81	Enoxaparin
Saketh R. Guntupalli [23]	RCT	58.0 ± 16.5	58.5 ± 17.0	204 F	196 F	400	204	196	Enoxaparin
Mostafa El Mokadem [24]	RCT	61.26 ± 11.23	59.94 ± 9.71	20 (40%) M/30 (60%) F	22(44%) M/28(56%) F	100	50	50	Enoxaparin

**Table 3:** Disease characteristics across included studies.

Author	Malignancy Site (Intervention)	Malignancy Site (Comparator)	Cancer Type	Cancer Stage	Diagnosis	VTE (Intervention)	VTE (Comparator)	Follow-Up
Robert D. McBane II et al. [19]	Brain (2.068%), Breast (11.03%), Colorectal (12.41%), ENT (2.06%), GU (8.96%), Gynecologic (9.655%), Lung (22.06%), Melanoma (0), Neuroendocrine (1.379%), Pancreatic/Hepatobiliary (15.86%), Sarcoma (2.068%), Thyroid (0), Upper GI (4.827%), Other (0)	Brain (3.52%), Breast (8.5%), Colorectal (20.4%), ENT (0.70%), GU (9.85%), Gynecologic (10.56%), Lung (13.38%), Melanoma (2.82%), Neuroendocrine (2.11%), Pancreatic/Hepatobiliary (16.90%), Sarcoma (0.70%), Thyroid (0.70%), Upper GI (2.81%), Other (0.70%)	Solid tumors (majority): lung, colorectal, pancreatic/hepatobiliary, gynecologic, breast; hematological malignancies (leukemia, lymphoma, myeloma) also included	Metastatic disease: apixaban 65.3%, dalteparin 66.0%	A recurrent DVT—duplex ultrasonography, venography, CT, or MRI. A recurrent PE-CT, MR, conventional pulmonary angiography, or VQ imaging	PE only: 43.5%; PE + DVT: 11.6%; DVT only: 36.7%; lower extremity DVT: 31.3%; upper extremity DVT: 17.0%; splanchnic VT: 8.2%; cerebral VT: 1.4%	PE only: 38.5%; PE + DVT: 12.2%; DVT only: 35.1%; lower extremity DVT: 33.8%; upper extremity DVT: 14.2%; splanchnic VT: 18.2%; cerebral VT: 0%	6 months
Giancarlo Agnelli et al [20].	Gastrointestinal Cancer—32.5%, Lung Cancer—17.3%, Breast Cancer—13.4%, Genitourinary Cancer—12%, Gynecologic Cancer—10.3%, Hematological Malignancies—7.4%, Others—6.9%	Same distribution (small group; balanced between arms)	Mixed solid tumors (dominant) + hematologic malignancies	Locally advanced or metastatic cancer in majority; >70% had metastasis	Objective imaging: CT, ultrasound, perfusion scan for VTE confirmation	Symptomatic or incidental PE, proximal DVT or both	Symptomatic or incidental PE, proximal DVT or both	6 months for treatment, outcomes monitored until 20th day
Jwa HoonKim et al. [21]	Gastric (45.2%), Pancreatic (23.8%), Biliary Tract (16.6%), Esophageal (7.1%), Ampulla of Vater (7.1%)	Similar distribution (exact numbers not differentially reported; trial groups balanced)	Upper gastrointestinal, hepatobiliary, pancreatic cancers only	Advanced cancer in 86–88% (majority stage III–IV, metastatic or unresectable)	CT, ultrasonography or perfusion scan	PE (dominant) & DVT-DOAC subgroup: 79.5% PE, 20.5% DVT; symptomatic & incidental included	Dalteparin: 69.6% PE, remainder DVT; symptomatic & incidental	6 months
G. AGNELLI [22]	Prostate (15.9%), Breast (14.8%), Colon (12.5%), Bladder (8%), Lung (8%)	Same distribution (small group; balanced between arms)	Mixed solid tumors	~33% metastatic; active cancer = 3.1%; history of cancer = 6.8%	Objective imaging: symptomatic proximal DVT or PE	DVT/PE ratio (Api:Eno)—2.03:1, Proximal DVT with clots in femoral, common femoral or iliac veins (Api:Eno)—(79.3:82.4), intermediate or extensive PE (Api:Eno)—(83.3:77.3), indicating apixaban: DVT ≈ 67%, PE ≈ 33%; comparator: DVT ≈ 73%, PE ≈ 27%	Referred in intervention arm in ratios	6 months
Saketh R. Guntupalli [23]	Uterine (39.7%), Ovarian and Fallopian (44.1%), Cervical (9.8%), Vulvar or Vaginal (3.4%), Other (2.9%).	Uterine (42.3%), Ovarian and Fallopian (39.8%), Cervical (11.2%), Vulvar or Vaginal (3.6%), Other (3.1%).	Gynecologic cancers (uterine, ovarian/fallopian tube, cervical, vulvar/vaginal)	Low stage (I or II) (Api vs. Eno)—80 (39.2%) vs. 85 (43.4%); high (III or IV) (Api vs. Eno)—84 (41.2%) vs. 74 (37.8%)	Well’s criteria for DVT, confirmed by Doppler and CT	2 VTE events (1%): 0 DVT, 2 PE (1%)	3 VTE events (1.5%): 3 DVT, 0 PE	90 days
Mostafa El Mokadem [24]	Colon (46%), Bladder (8%), Prostate (12%), Liver (4%), Ovary (10%), Uterus (6%), Breast (14%)	Colon (38%), Bladder (4%), Prostate (10%), Liver (8%), Ovary (12%), Uterus (16%), Breast (8%)	Mixed solid tumors—colon most common (42%)	Cancer stage I (Api/Eno) 6%/2%, cancer stage II (Api/Eno)—14%/10%, cancer stage IV (Api/Eno)—80%/88%	Venous Doppler ultrasound	Iliofemoral DVT (38%), femoropopliteal DVT (34%), calf vein thrombosis (20%), upper limb DVT (8%), associated thrombophlebitis (14%)	Iliofemoral DVT (48%), femoropopliteal DVT (32%), calf vein thrombosis (18%), upper limb DVT (2%), associated thrombophlebitis (10%)	6 months

## Data Availability

All data supporting the findings of this study are included within the article and its Supplementary Materials. No new datasets were generated or analyzed beyond those included in the published studies.

## Supplementary Materials

The following supporting information can be downloaded at: https://www.mdpi.com/article/10.3390/jcm15145341/s1, File S1: PRISMA checklist [31]; Figure S1: Funnel plot for recurrent VTE; Figure S2: Funnel plot for Bleeding; Figure S3: Funnel for Clinically relevant Non -Major bleeding (CRNMB).
