# Supplementary material for: Apixaban Versus Low-Molecular-Weight Heparin for Cancer-Associated Venous Thromboembolism: A Systematic Review and Meta-Analysis
Source: J Clin Med. 2026 Jul 8;15(14):5341. doi: 10.3390/jcm15145341 (PMC13410163; doi:10.3390/jcm15145341)
Supplement: Supplementary file 1 [file jcm-15-05341-s001.zip › Supplementary material.pdf]

**Apixaban Versus Low-Molecular-Weight Heparin for Cancer-Associated Venous Thromboembolism: A Systematic Review and Meta-Analysis**

Sumit Aggarwal [1*#] , Vikram Singh [1*], Aayushi Bhasin [1*], Sachit Anand, Heena Tabassum[#]

Indian Council of Medical Research, New Delhi

All India Institute of Medical Sciences, New Delhi

*Authors contributed equally

[#]Corresponding author

Email- drsumiticmr@gmail.com , drheenaicmr@gmail.com

Research question: **"What is the comparative efficacy and safety of apixaban versus other LMWH (Low molecular weight heparin, particularly Dalteparin and enoxaparin) in the treatment of cancer-associated venous thromboembolism?"**

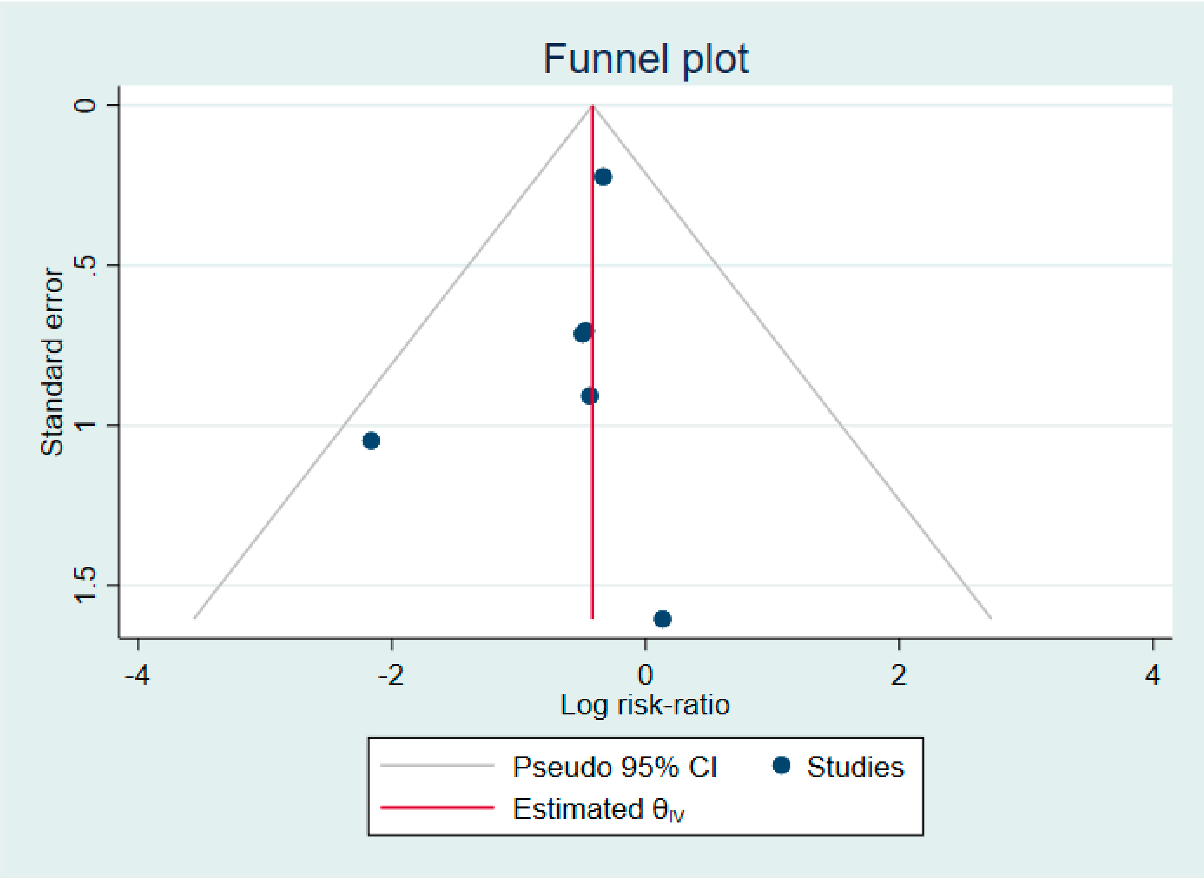


**Figure S1 : Funnel plot for recurrent VTE**

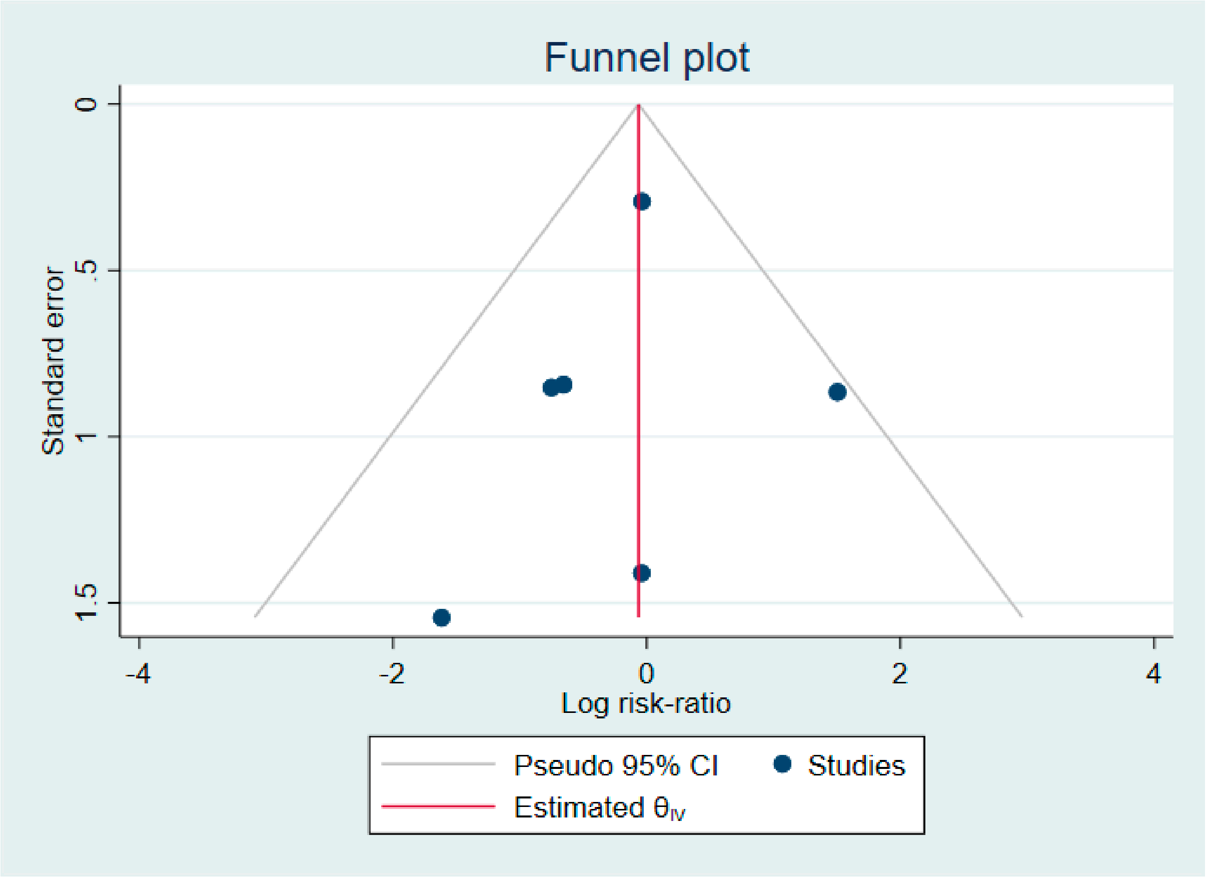


**Figure S2: Funnel plot for Bleeding**

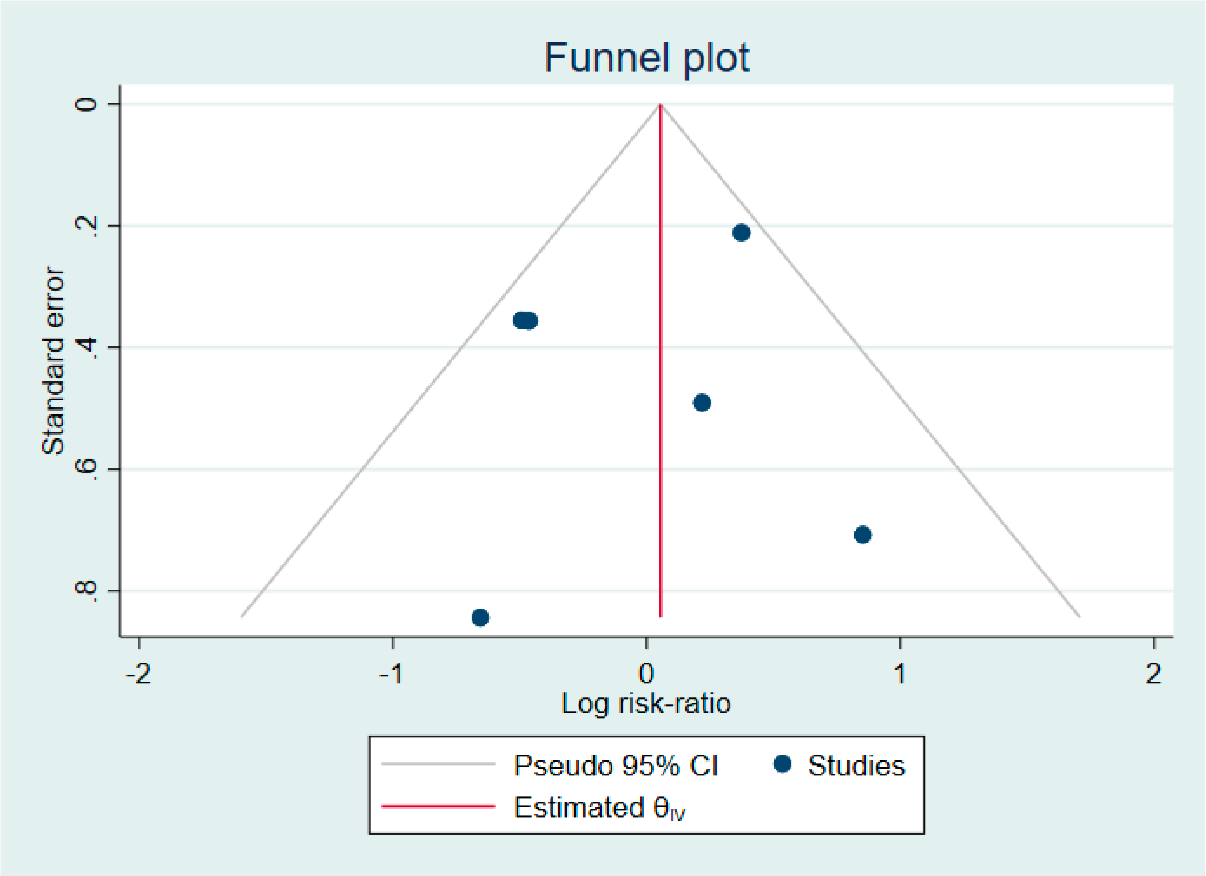


**Figure S3 : Funnel for Clinically relevant Non -Major bleeding (CRNMB)**
